# Computer-aided design and fabrication of nasal prostheses: a semi-automated algorithm using statistical shape modeling

**DOI:** 10.1007/s11548-024-03206-y

**Published:** 2024-06-06

**Authors:** T. Bannink, M. de Ridder, S. Bouman, M. J. A. van Alphen, R. L. P. van Veen, M. W. M. van den Brekel, M. B. Karakullukçu

**Affiliations:** 1 Department of Head and Neck Oncology and Surgery, Netherlands Cancer Institute—Antoni van Leeuwenhoek, Amsterdam, The Netherlands; 2 Department of Head and Neck Oncology and Surgery, Netherlands Cancer Institute—Antoni van Leeuwenhoek, Verwelius 3D Lab, Amsterdam, The Netherlands; 3 Amsterdam Center of Language and Communication, University of Amsterdam, Amsterdam, The Netherlands; 4 Department of Oral and Maxillofacial Surgery, Amsterdam University Medical Center, Amsterdam, The Netherlands

**Keywords:** Nasal prosthesis, Computer-aided design, Statistical shape modeling, Morphable model, 3D printing

## Abstract

**Purpose:**

This research aimed to develop an innovative method for designing and fabricating nasal prostheses that reduces anaplastologist expertise dependency while maintaining quality and appearance, allowing patients to regain their normal facial appearance.

**Methods:**

The method involved statistical shape modeling using a morphable face model and 3D data acquired through optical scanning or CT. An automated design process generated patient-specific fits and appearances using regular prosthesis materials and 3D printing of molds. Manual input was required for specific case-related details.

**Results:**

The developed method met all predefined requirements, replacing analog impression-making and offering compatibility with various data acquisition methods. Prostheses created through this method exhibited equivalent aesthetics to conventionally fabricated ones while reducing the skill dependency typically associated with prosthetic design and fabrication.

**Conclusions:**

This method provides a promising approach for both temporary and definitive nasal prostheses, with the potential for remote prosthesis fabrication in areas lacking anaplastology care. While new skills are required for data acquisition and algorithm control, these technologies are increasingly accessible. Further clinical studies will help validate its effectiveness, and ongoing technological advancements may lead to even more advanced and skill-independent prosthesis fabrication methods in the future.

## Introduction

Loss of the nose due to trauma or disease profoundly impacts an individual’s physical appearance and psychological well-being. People with facial differences regularly encounter unwanted attention, such as staring, questions, and comments in everyday social interactions [1]. Reconstruction of the face with a nasal prosthesis revokes the appearance-altering effects of the amputation. It enables patients to ‘pass as normal’ again and regain their anonymity in public [1–3].

Achieving a normal facial appearance requires seamless prosthesis integration with the face, encompassing secure attachment, comfort, and harmonious color, texture, and shape [2, 3]. In the traditional fabrication method, all of this is accomplished by hand, making it time-consuming and skill-dependent. Particularly in nasal prostheses, the shape strongly depends on the skill and expertise of the anaplastologist by missing a template as available in prosthesis design of paired structures. An innovative method that overcomes this would be valuable by making the design and fabrication of nasal prostheses less expertise-dependent.

Advanced 3D techniques offer opportunities to create a template for facilitating nasal prosthesis design. Research shows the potential of digitized protocols in anaplastology using imaging and computer-aided design and manufacturing (CAD-CAM) [4–6]. Approaches vary, often using pre-operative photographic or imaging data or a nose database [7–11]. However, imaging of the previous anatomy is often unavailable or extensively distorted. A nose database can help by offering a starting point for prosthesis design. However, previously described databases are not readily accessible [4, 11]. Therefore, Jablonski et al. [11] recently generated a new open-access database of 44 nose models based on a morphable face model (MFM) approach. This database may support anaplastologists by supplying a template. Yet model selection, positioning, modification, and processing into a 3D printed model or mold requires an understanding of prosthetic principles and CAD techniques. An overall decrease in skill dependency in nasal prosthesis fabrication could be achieved by an alternative, more advanced application of an MFM.

In 2005, Basso and Vetter [12] presented a method to automatically reconstruct missing areas of a face scan by using an MFM [13]. An MFM is a statistical model of a dataset of 3D face scans, which can generate new faces by combining the exemplar faces. In 2011, Mueller et al. [14] applied an MFM to a patient’s face scan after total rhinectomy to produce a face model statistically consistent with the unaffected part of the patient’s face. Based on the reconstruction, a nasal prosthesis was 3D printed. This research highlights the potential of using an MFM in nasal prosthesis design, as well as areas for improvement regarding the fit and appearance of the prosthesis. This was partly related to the direct printing approach, which has yet to produce prostheses of equal quality as those traditionally fabricated [15–20]. Nowadays, an improved version of the used MFM, the Basel Face Model (BFM-2019), is publicly available for utilization [21].

This study aimed to develop a new MFM-based method for designing and fabricating nasal prostheses. We combined a semi-automated design algorithm to reduce expertise dependency with an indirect 3D printing approach to enable regular prosthesis materials for optimal appearance.

## Methods

The workflow was developed based on the following requirements to ensure an effective, widely applicable method.

1. The workflow obviates the need for analog impression-making.
2. The workflow is compatible with optical scanning and computer tomography (CT).
3. The workflow utilizes automatic shape modeling.
4. The workflow is compatible with regular prosthesis materials.

### Overview

Digital nasal prosthesis design is based on two key elements: a 3D mesh of the patient’s affected face and a surface model of the intended nasal shape. Subtraction of the patient’s face model (FM) from the nose model (NM) results in a prosthesis model (PM) with a patient-specific fit to the skin surface. In this method, the NM is generated by an algorithm based on statistical shape modeling. An MFM generates a face model with maximum correspondence to the FM. Cropping this fitted model to the nasal area results in the NM. The NM was imported in a second algorithm, which converted the NM into the PM and the corresponding 3D printable mold. The printed mold was packed with skin-tone-colored silicone, resulting in the nasal prosthesis.

### Image acquisition

The 3D surface data of the patient’s face was acquired using the Artec Space Spider optical scanner (Artec 3D, Luxembourg). The facial morphology was captured by moving the handheld scanner around the patient’s face at 20–30 cm. The acquisition took around two minutes. Post-processing of the scan data was done in Artec Studio 16 Professional, resulting in a high-resolution surface model (sharp fusion with 3D resolution set at 0.3 mm). Patients should be able to tolerate flashes of light to be scanned, and they should remove facial hair. If optical scanning was not possible or available, the surface model could be obtained from CT data.

### Statistical shape modeling

**Fig. 1:**
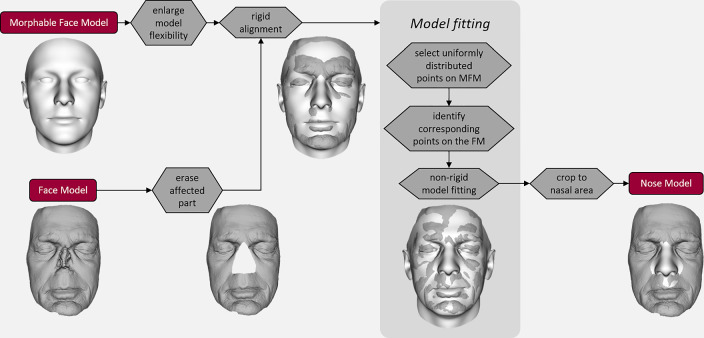
Computation of the nose model (NM) using statistical shape modeling. First, the affected part of the patient’s face is erased from the face model (FM). Next, the FM and morphable face model (MFM) are aligned. Model fitting results in an NM, statistically consistent with the FM

The FM was used to compute the NM. An algorithm was drawn up based on Scalismo,^1^ a library for statistical shape modeling in Scala, developed by the Graphics and Vision Research Group at the University of Basel. This algorithm automatically matches an MFM with the patient’s FM by adjusting the model parameters until the optimal reconstruction of the patient’s face is achieved. For this reconstruction to have a normal appearance, the affected part of the patient’s face was erased from the input data, preventing it from affecting the morphing procedure. As MFM, the BFM-2019 [21] was used, a publicly available database including face scans of 100 males and 100 females, mostly Europeans between 18 and 80 years old [22]. To enable the reconstruction of shape variations that differ from the data set, the model flexibility was enlarged by adding general smooth deformation fields [23]. Figure 1 shows an overview of the statistical shape modeling algorithm.

### Prosthesis model and mold

**Fig. 2:**
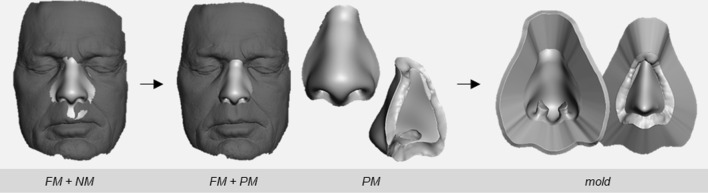
Processing the nose model (NM) to the prosthesis model (PM) and the corresponding two-part mold

The FM and NM were opened together in Meshmixer 3.5 (Autodesk Inc., CA, USA). By subtracting the FM from the NM, the PM was created, and this was converted in the corresponding mold (Fig. 2). A second algorithm was drawn up in Python 2.7 (Python Software Foundation) using the mm-API library^2^ to control the Meshmixer desktop application remotely. With this API (application programming interface) algorithm, the required operations inside Meshmixer were executed automatically. The code included the following protocol and associated operations and settings:

#### Prosthesis model

1. Increase the NM mesh density for more accurate processing into PM.
2. Define the contour of the prosthesis and create a smooth transition between the NM and FM by sculpting the border of the NM inward of the FM.
3. Set a wall thickness of the NM of 6 mm.
4. Smooth the inner surface of the NM to facilitate cleaning of the prosthesis.
5. Subtract the FM from the NM to create the PM with a seamless connection to the FM.
6. Smooth the connecting surface of the PM for comfortable attachment.
7. Sculpt the nostrils.

#### Mold

8. Separate the inner and outer shells of the PM to create the two-part mold.
9. Stretch the border of both shells with 0.3 mm offset in between to ensure tight mold closure.
10. Set a wall thickness of both shells of 4 mm.
11. Export the two mold parts for 3D printing.

Steps 2 and 7 require manual input because of the case-specific nature of these operations. The algorithm pauses and instructs the anaplastologist.

### 3D printing

**Fig. 3:**
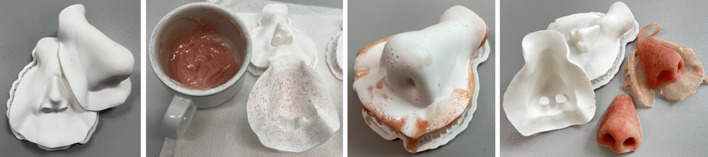
Prosthesis fabrication. **a** 3D printed mold. **b** Skin-tone-colored silicone and the anterior part of the mold after sprinkling microfibers. **c** The closed mold after filling with silicone. **d** When cured, the prosthesis was released from the mold, and excess silicone was removed

The mold was 3D printed using a stereolithography Form 3B desktop printer (Formlabs Inc., MA, USA). After testing multiple resins, Rigid 10 K was the most suitable Formlabs resin, which did not affect the silicone. Rigid 10 K is a highly stiff (ultimate tensile strength of 65 MPa, tensile modulus of 10 GPa) resin with a smooth matte finish (Fig. 3a). For printing, the mold parts were positioned in such an orientation that scaffolds were only positioned at the outside of the mold, so no traces of the scaffolds will be visible on the prosthesis. Post-processing was done following the manufacturer’s instructions: 20 min of washing in isopropanol and 60 min of curing at 70 °C.

### Prosthesis fabrication

The protocol for prosthesis fabrication with the 3D printed mold was similar to the traditional plaster molds. First, the mold was layered with dish soap to facilitate the release of the prosthesis. Red and brown microfibers were sprinkled on the mold surface (Fig. 3b) to provide the increased appearance of depth and light scattering and to simulate the effect of capillary blood vessels. The mold was filled with silicone (VST-30; Factor II Inc., AZ, USA), colored to match the patient’s skin tone, and closed (Fig. 3c). The prosthesis was released from the mold after at least four hours of curing at room temperature. After trimming the prosthesis edge and opening the nostrils, the prosthesis was ready to wear (Fig. 3d).

## Results

The developed method for nasal prosthesis design and fabrication met all set requirements. The analog impression, traditionally used to position the prosthesis and create a seamless fit, was replaced by the digital model of the patient’s face (req. 1). This face model was obtained using optical scanning or CT (req. 2). Determining the nasal shape was fully automated by the statistical shape modeling algorithm (req. 3). Also, the connection to the skin was created automatically as well as conversion of the PM to the 3D printable mold. Only defining the outline of the prosthesis, creating a smooth transition between the prosthesis and the skin surface, and sculpting the nostrils had to be done manually because of their case-specific nature. Lastly, the method worked successfully with the regularly used prosthesis materials (req. 4).

**Fig. 4:**
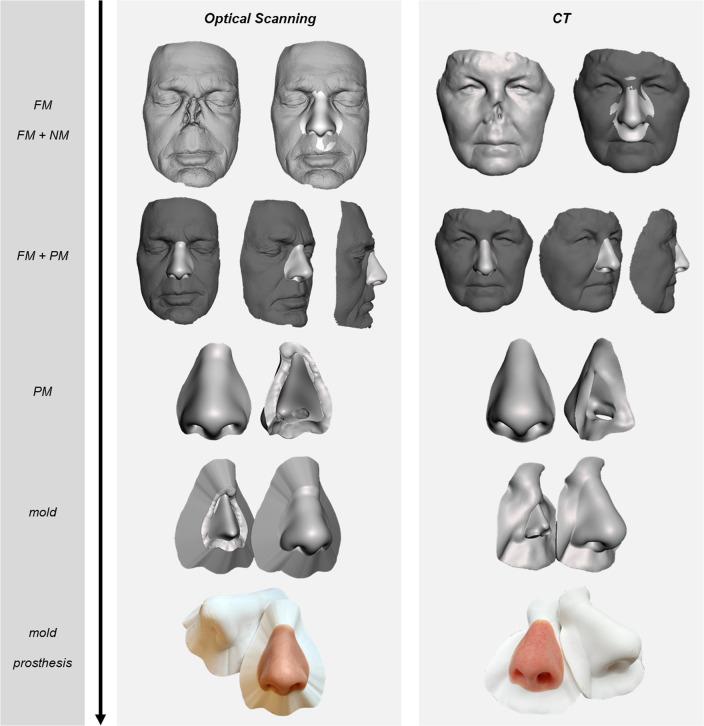
From top to bottom, for two cases, the nasal prosthesis design and fabrication process following the described method. On the left side is a case based on surface data acquired by optical scanning (3D resolution 0.3 mm). On the right side, a case based on CT data (pixel spacing 0.46 mm, slice thickness 1 mm)

The method resulted in nasal prostheses with a patient-specific shape based on the patient’s facial morphology. Figure 4 illustrates two cases based on FMs obtained by optical scanning and CT. Based on the FMs, the statistical shape modeling algorithm successfully fitted the MFM, which resulted in NMs statistically consistent with the FMs. The second algorithm created the PMs and corresponding molds. Stereolithography printing resulted in two-part molds with smooth surfaces, which were used for fabricating the prostheses without polishing. By visual inspection, the resulting prostheses showed equivalent appearances to conventionally manufactured prostheses that were available from other patients.

## Discussion

In this study, we developed a new method for nasal prosthesis design that supports the anaplastologist in providing these patients with prostheses consistent with their facial morphology. Automating the design using statistical shape modeling, surface scanning, and indirect printing enabled us to facilitate prosthesis design. The developed method met all predetermined requirements. The cases presented in this study demonstrate the potential of the method to create nasal prostheses with a patient-specific shape and fit based on the patient’s facial morphology while reducing expertise dependency.

We were able to set up our method thanks to the materials published by the Graphics and Vision Research Group of the University of Basel. A series of publications includes the development of a method for statically motived 3D face reconstruction using a morphable model [12, 13, 21]. Mueller et al. [14] were in 2011 the first to apply this reconstruction method for nasal prosthesis design. Recently, Jablonski et al. [11] used an MFM to create a nose model database to facilitate nasal prosthesis design. We added a new method that focuses on the automation of the entire design process, and we combined this with an indirect printing approach for improved prosthesis appearance. To what extent prostheses resulting from the described method succeed in optimally restoring the appearance of the patient’s face will emerge from a clinical study, the results of which will follow shortly.

The new method reduces the dependency on the skill and experience of the anaplastologist because an automated design algorithm generates the shape. However, using technology also requires new skills, such as obtaining the surface data and controlling the algorithms. Fortunately, due to the rapid development of these technologies and, therefore, availability, this has become very accessible. We designed algorithms that make the workflow as easy as possible. Every standard procedure was automated. Only the patient-specific ones must be carried out manually when an instruction appears.

Fabricating the prosthesis by filling the 3D printed mold was done in the same way as in the traditional method and with regular materials. Therefore, the appearance and quality of the prostheses are equal to traditionally fabricated prostheses. 3D printing of the prosthesis itself could further reduce the skill dependency of prosthesis fabrication. However, the aesthetic appearance of 3D printed prostheses is less due to the different nature of the used printing materials and the limitations in the coloring of the prosthesis [15–19]. Unkovskiy et al. [20] successfully printed an auricular prosthesis of medical-grade silicone with various flexibility grades. Time-consuming analog post-processing, including extrinsic coloring of the prosthesis, resulted in a reasonable aesthetic outcome but still less than one conventionally created. Until technical advances allow printing clinically applicable prostheses directly, printing the mold instead of the prosthesis provides a better balance of innovation and traditional craftsmanship. In addition, the mold can also be used to cast the wax pattern instead of the silicone prosthesis. This allows the anaplastologist to maintain the fitting session as done in the traditional method and, together with the patient, to fine-tune the prosthetic model as desired.

Although the described technique can be used for definitive prostheses, it can also be used for temporary prostheses. Patients often have to wait for several months before prosthesis fabrication can start and the time-consuming process is finished. If equipment is available, this technique is a fast and cheap solution to provide patients with a prosthesis shortly after surgery. Another application of the technique can be remote prosthesis fabrication for patients living in areas where anaplastology care is unavailable. The only required input, surface data of the face, could be provided by scanning the face with a 3D camera, currently available in most smartphones.

## Conclusion

This research presents an innovative method for designing and fabricating nasal prostheses. The described method represents an alternative to traditional fabrication methods based on an analog impression and manual sculpting of the prosthesis model. The new approach combines 3D data acquired through optical scanning or CT with statistical shape modeling, offering a patient-specific fit and appearance. While it reduces dependency on the skills and experience of the anaplastologist, it requires new skills in data acquisition and algorithm control. The technique can serve temporary and definitive prosthesis needs, and remote application may benefit areas with limited anaplastology care. Clinical studies will help validate effectiveness, and ongoing technological advancements may further improve the process in the future.

## Footnotes

1 https://scalismo.org/.

2 https://github.com/meshmixer/mm-api.

## References

[1] Yaron G, Meershoek A, Widdershoven G, Slatman J (2018) Recognizing difference: in/visibility in the everyday life of individuals with facial limb absence. Disabil Soc 33(5):743–762. 10.1080/09687599.2018.1454300

[2] Yaron G, Meershoek A, Widdershoven G, van den Brekel M, Slatman J (2017) Facing a disruptive face: embodiment in the everyday experiences of “disfigured” individuals. Hum Stud 40(2):285–307. 10.1007/s10746-017-9426-8

[3] Wondergem M, Lieben G, Bouman S, van den Brekel MW, Lohuis PJ (2016) Patients’ satisfaction with facial prostheses. Br J Oral Maxillofac Surg 54(4):394–399. 10.1016/j.bjoms.2015.09.011 26508540 10.1016/j.bjoms.2015.09.011

[4] Farook TH, Jamayet NB, Abdullah JY, Rajion ZA, Alam MK (2020) A systematic review of the computerized tools and digital techniques applied to fabricate nasal, auricular, orbital and ocular prostheses for facial defect rehabilitation. J Stomatol Oral Maxillofac Surg 121(3):268–277. 10.1016/j.jormas.2019.10.003 31610244 10.1016/j.jormas.2019.10.003

[5] Cristache CM, Tudor I, Moraru L, Cristache G, Lanza A, Burlibasa M (2021) Digital workflow in maxillofacial prosthodontics—an update on defect data acquisition, editing and design using open-source and commercial available software. Appl Sci 11(3):973. 10.3390/app11030973

[6] Tanveer W, Ridwan-Pramana A, Molinero-Mourelle P, Koolstra JH, Forouzanfar T (2021) Systematic review of clinical applications of cad/cam technology for craniofacial implants placement and manufacturing of nasal prostheses. Int J Environ Res Public Health 18(7):3756. 10.3390/ijerph18073756 33916853 10.3390/ijerph18073756 PMC8038514

[7] Reitemeier B, Götzel B, Schöne C, Stockmann F, Müller R, Lexmann J, Meissner H (2013) Creation and utilization of a digital database for nasal prosthesis models. Onkologie 36(1–2):7–11. 10.1159/000346668 23429325 10.1159/000346668

[8] Fantini M, De Crescenzio F, Ciocca L (2013) Design and rapid manufacturing of anatomical prosthesis for facial rehabilitation. Int J Interact Des Manuf (IJIDeM) 7(1):51–62. 10.1007/s12008-012-0159-7

[9] Palousek D, Rosicky J, Koutny D (2014) Use of digital technologies for nasal prosthesis manufacturing. Prosthet Orthot Int 38(2):171–175. 10.1177/0309364613489333 23798039 10.1177/0309364613489333

[10] Unkovskiy A, Roehler A, Huettig F, Geis-Gerstorfer J, Brom J, Keutel C, Spintzyk S (2019) Simplifying the digital workflow of facial prostheses manufacturing using a three-dimensional (3d) database: setup, development, and aspects of virtual data validation for reproduction. J Prosthodont Res 63(3):313–320. 10.1016/j.jpor.2019.01.004 30792148 10.1016/j.jpor.2019.01.004

[11] Jablonski RY, Malhotra T, Coward TJ, Shaw D, Bojke C, Pavitt SH, Nattress BR, Keeling AJ (2023) Digital database for nasal prosthesis design with a 3d morphable face model approach. J Prosthet Dent. 10.1016/j.prosdent.2023.02.019 37019749 10.1016/j.prosdent.2023.02.019

[12] Basso C, Vetter T (2005) Statistically motivated 3d faces reconstruction. In: Proceedings of the 2nd International Conference on Reconstruction of Soft Facial Parts. Luchterhand Publishers, Remagen

[13] Blanz V, Vetter T (1999) A morphable model for the synthesis of 3d faces. In: Proceedings of the 26th annual conference on Computer graphics and interactive techniques. ACM Press/Addison-Wesley Publishing Co. 10.1145/311535.311556

[14] Mueller AA, Paysan P, Schumacher R, Zeilhofer HF, Berg-Boerner BI, Maurer J, Vetter T, Schkommodau E, Juergens P, Schwenzer-Zimmerer K (2011) Missing facial parts computed by a morphable model and transferred directly to a polyamide laser-sintered prosthesis: an innovation study. Br J Oral Maxillofac Surg 49(8):e67-71. 10.1016/j.bjoms.2011.02.007 21458119 10.1016/j.bjoms.2011.02.007

[15] Zardawi FM, Xiao K, Noort Rv, Yates JM (2015) Mechanical properties of 3d printed facial prostheses compared to silicone polymer prostheses. Eur Sci J ESJ 11(12)

[16] Xiao K, Wuerger SM, Mostafa F, Sohaib A, Yates JM (2016) Colour image reproduction for 3d printing facial prostheses. In: Shishkovsky IV (ed) New trends in 3d printing*,* IntechOpen

[17] Unkovskiy A, Spintzyk S, Brom J, Huettig F, Keutel C (2018) Direct 3d printing of silicone facial prostheses: a preliminary experience in digital workflow. J Prosthet Dent 120(2):303–308. 10.1016/j.prosdent.2017.11.007 29429837 10.1016/j.prosdent.2017.11.007

[18] Nuseir A, Hatamleh MMd, Alnazzawi A, Al-Rabab’ah M, Kamel B, Jaradat E (2019) Direct 3d printing of flexible nasal prosthesis: Optimized digital workflow from scan to fit. J Prosthodont 28(1):10–14. 10.1111/jopr.13001 30461125 10.1111/jopr.13001

[19] Abdullah AM, Mohamad D, Din TNDT, Yahya S, Akil HM, Rajion ZA (2019) Fabrication of nasal prosthesis utilising an affordable 3d printer. Int J Adv Manuf Technol 100(5):1907–1912. 10.1007/s00170-018-2831-y

[20] Unkovskiy A, Wahl E, Huettig F, Keutel C, Spintzyk S (2020) Multimaterial 3d printing of a definitive silicone auricular prosthesis: An improved technique. J Prosthet Dent. 10.1016/j.prosdent.2020.02.021 32680736 10.1016/j.prosdent.2020.02.021

[21] Gerig T, Morel-Forster A, Blumer C, Egger B, Lüthi M, Schönborn S, Vetter T (2018) Morphable face models - an open framework*.* In: 2018 13th IEEE International Conference on Automatic Face & Gesture Recognition (FG 2018), pp 75–82. 10.1109/FG.2018.00021

[22] Paysan P, Knothe R, Amberg B, Romdhani S, Vetter T (2009) A 3d face model for pose and illumination invariant face recognition*.* In: 2009 Sixth IEEE International Conference on Advanced Video and Signal Based Surveillance, pp 296–301. 10.1109/AVSS.2009.58

[23] Lüthi M, Gerig T, Jud C, Vetter T (2018) Gaussian process morphable models. IEEE Trans Pattern Anal Mach Intell 40(8):1860–1873. 10.1109/TPAMI.2017.2739743 28816655 10.1109/TPAMI.2017.2739743

